# Vitamin D supplementation for nonalcoholic fatty liver disease in type 2 diabetes mellitus: A protocol for a systematic review and meta-analysis

**DOI:** 10.1097/MD.0000000000020148

**Published:** 2020-05-08

**Authors:** Shengju Wang, Baochao Cai, Xuke Han, Yang Gao, Xiaoran Zhang, Ruili Wang, Yuan Zhang, Qiu Chen

**Affiliations:** a Department of Endocrinology, Hospital of Chengdu University of Traditional Chinese Medicine, Chengdu 610072; b Endocrinology Department, Jiaxing Hospital of Traditional Chinese Medicine, Jiaxing 314001; c Diabetes Department, Jintang County Traditional Chinese Medicine Hospital, Chengdu 610400, PR China.

**Keywords:** meta-analysis, nonalcoholic fatty liver disease, protocol, type 2 diabetes mellitus, vitamin D supplementation

## Abstract

**Background:**

Nonalcoholic fatty liver disease (NAFLD) is strongly associated with type 2 diabetes mellitus (T2DM), and low vitamin D levels are positively associated with NAFLD and T2DM. But there is absence of convincing evidence-based medicine to confirm the efficacy of vitamin D supplementation for T2DM with NAFLD. Thus, we aimed to conduct this meta-analysis to summarize the efficacy of vitamin D supplementation for T2DM combined with NAFLD, and help to further clarify its beneficial action on diabetic patients with NAFLD.

**Methods:**

The study only selects clinical randomized controlled trials of vitamin D supplementation for T2DM combined with NAFLD. We will search each database from the built-in until July 2020. The English literature mainly searches Cochrane Library, Pubmed, EMBASE, and Web of Science. While the Chinese literature comes from CNKI, CBM, VIP, and Wangfang database. Meanwhile, we will retrieve clinical trial registries and grey literature. Two researchers worked independently on literature selection, data extraction, and quality assessment. The dichotomous data is represented by relative risk (RR), and the continuous is expressed by mean difference (MD) or standard mean difference (SMD), eventually the data is synthesized using a fixed effect model (FEM) or a random effect model (REM) depending on the heterogeneity. The imaging markers of liver, biomarkers of hepatic steatosis, serological indexes of hepatic fibrosis, serum NAFLD liver fat score were evaluated as the main outcomes. While several secondary outcomes were also evaluated in this study. The statistical analysis of this meta-analysis was conducted by RevMan software version 5.3.

**Results:**

This meta-analysis will further determine the beneficial efficacy of vitamin D supplementation for T2DM combined with NAFLD.

**Conclusion:**

This study determines the positive efficacy of vitamin D supplementation for diabetic patients with NAFLD.

## 1 Introduction

Nonalcoholic fatty liver disease (NAFLD) is a spectrum of fat-associated liver conditions, may progress to nonalcoholic steatohepatitis (NASH), fibrosis, and cirrhosis.^[1]^ The insulin resistance (IR), oxidative stress, and inflammation are major contributions for the development of NAFLD.^[2,3]^ It has become the major cause of chronic liver disease worldwide.^[4–6]^ The epidemic proportion of NAFLD is increasing, it has been estimated to be 25% to 30% among general adults in developed countries.^[7,8]^ NAFLD is strongly associated with metabolic syndrome (MS) or type 2 diabetes mellitus (T2DM).^[9]^ It has been reported that the prevalence of NAFLD in T2DM is up to 70%.^[7,10]^ T2DM and IR are surely the strongest predictors of the progression of NAFLD to advanced fibrosis and cirrhosis.^[11,12]^ Several published articles have manifested that NAFLD substantially increased the risk of incident T2DM.^[13–16]^ Since IR is a key driver of NAFLD and T2DM, insulin-sensitizing drugs have been used to the NAFLD and T2DM treatment.^[17]^ However, the absence of substantial clinical evidence demonstrated that insulin-sensitizing drugs have major beneficial actions on the progression of NAFLD.

Vitamin D is a fat-soluble vitamin and regulates the bone homeostasis.^[18,19]^ Since vitamin D is an important component of many tissues, organs, and metabolic processes. The role of vitamin D has been extended to a wide-ranging non-skeletal health problems,^[20]^ including MS, IR, T2DM, obesity, and cardiovascular disease.^[21–23]^ There is increasing epidemiological evidence implying that vitamin D deficiency is associated with increased risk of developing diabetes.^[24–26]^

Meanwhile, there has been a significant scientific interest in the relationship between vitamin D status and NAFLD. Vitamin D has numerous properties that modulate IR, fibrogenesis, chronic inflammation, suggesting that vitamin D may be a target for preventing the progression of NAFLD.^[27]^ Several clinical studies have shown that vitamin D deficiency may contribute to the onset and progression of NAFLD.^[28–30]^ But there are absent evidences whether the vitamin D supplementation is beneficial for diabetic patients with NAFLD. Thus, we intend to collect randomized controlled trials (RCTs) about vitamin D supplementation for T2DM combined with NAFLD based on evidence-based medicine, and conduct a meta-analysis of its efficacy to provide higher quality clinical evidence for vitamin D supplementation is beneficial for NAFLD in diabetic patients.

## 2 Methods

### 2.1 Protocol registration

The systematic review protocol has been registered on the INPLASY website (https://inplasy.com/inplasy-2020-3-0012/) and INPLASY registration number is INPLASY202030012. It is reported following the guidelines of Cochrane Handbook for Systematic Reviews of Interventions and the Preferred Reporting Items for Systematic Reviews and Meta-analysis Protocol (PRISM).^[31]^ If there are any adjustments throughout the study, we will fix and update the details in the final report.

### 2.2 Inclusion criteria

#### 2.2.1 Study design

The study only selects clinical randomized controlled trials of vitamin D supplementation for T2DM combined with NAFLD published in both Chinese and English. However, animal experiments, reviews, case reports, and non-randomized controlled trials are excluded.

#### 2.2.2 Participants

The patients with clinically diagnosed T2DM combined with NAFLD and treatment with vitamin D supplementation, regardless of race, gender, and age. NAFLD by other causes and patients with severe heart disease, liver and kidney dysfunction, mental illness, or a relevant drug allergic history will be not included.

#### 2.2.3 Interventions

Both groups were treated with standard diabetes treatments, including diet, exercise, hypoglycemic, and lipid-lowering therapies. The experiment group used vitamin D supplementation, while the control group applied for placebo, or no treatment. In addition, the 2 groups did not take any drugs that interfered with the outcome indicators. The follow-up time was ≥12 weeks.

#### 2.2.4 Outcomes

The primary outcomes include the improvement in clinical efficacy and imaging markers, biomarkers of hepatic steatosis, serological indexes of hepatic fibrosis, serum NAFLD liver fat score.

Secondary outcomes are mainly composed of fasting blood glucose, 2 hours postprandial blood glucose, HbA1c, serum insulin levels, body mass index (BMI), body weight, serological markers (LDL-cholesterol, triglyceride, HDL-cholesterol, aspartate transaminase (AST), alanine transaminase (ALT), γ-glutamyl transferase (GGT), albumin, etc), HOMA-IR, and adverse events.

### 2.3 Search methods

#### 2.3.1 Electronic searches

We will retrieve each database from the built-in until July 2020. The English literature mainly searches Cochrane Library, Pubmed, EMBASE, and Web of Science. While the Chinese literature comes from CNKI, CBM, VIP, and Wangfang database. We adopt the combination of heading terms and free words as search strategy which decided by all the reviewers. Search terms: vitamin D supplementation, vitamin D deficiency, 25-hydroxy vitamin D [25(OH)D], ergocalciferol(s), nonalcoholic fatty liver disease, nonalcoholic steatohepatitis, fatty liver, nonalcoholic fatty liver, liver fibrosis, liver cirrhosis, type 2 diabetes mellitus, type 2 diabetes, diabetes, diabetes mellitus. We will simply present the search process of the Cochrane Library, as shown in Table 1, adjusting different search methods according to different Chinese and English databases.

**Table 1:**
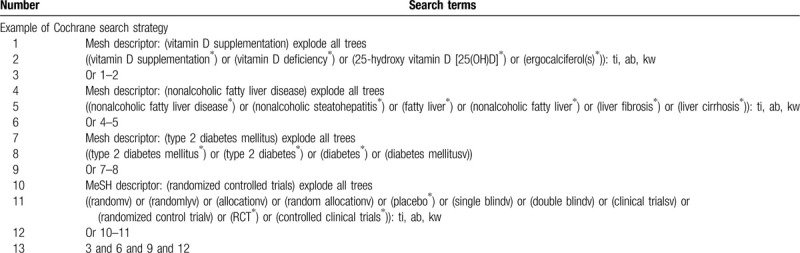
Cochrane Library search strategy.

#### 2.3.2 Searching other resources

At the same time, we will retrieve other resources to complete the deficiencies of the electronic databases, mainly searching for the clinical trial registries and grey literature about vitamin D for T2DM combined with NAFLD on the corresponding website.

### 2.4 Data collection and analysis

#### 2.4.1 Selection of studies

**Figure 1:**
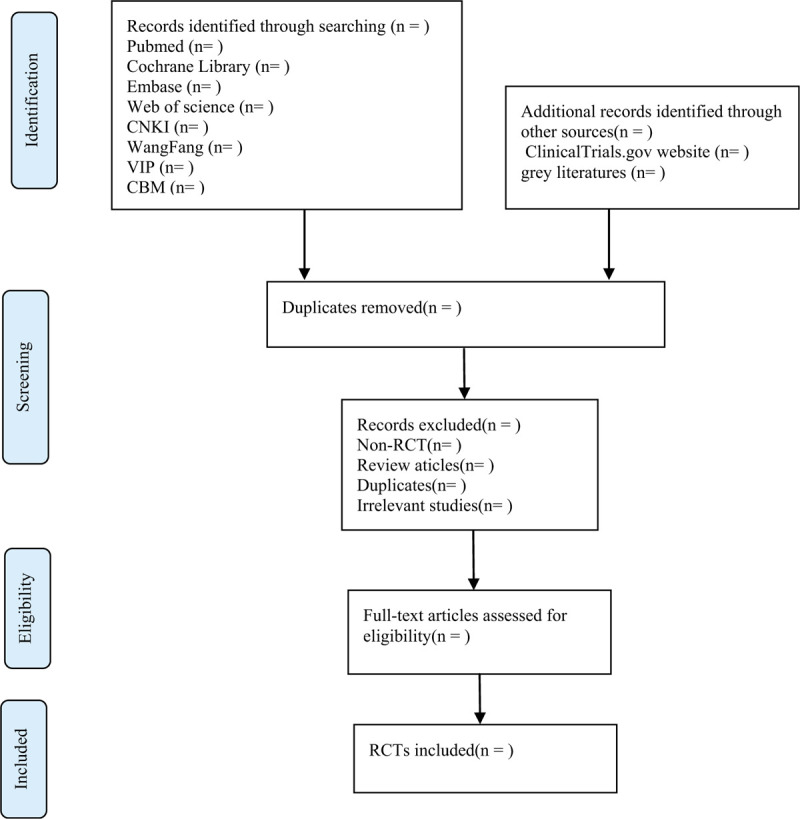
Flow chart of the study selection.

Import all literatures that meet the requirements into Endnote X8 software. Firstly, 2 independent reviewers initially screened the literatures that did not meet the pre-established standards of the study by reading the title and abstract. Secondly, download the remaining literatures and read the full text carefully to further decide whether to include or not. Finally, the results were cross-checked repeatedly by reviewers. If there is a disagreement in the above process, we can reach an agreement by discussing between both reviewers or seek an opinion from third party. PRISMA flow diagram (Fig. 1) will be used to show the screening process of the study.

#### 2.4.2 Data extraction and management

According to the characteristics of the study, we prepare an excel form for data collection before data extraction. Outcome indicators for eligible studies were independently extracted and filled in the data extraction form by 2 reviewers. If there is any argument, it can get an agreement by discussing through 2 reviewers or seek suggestions form third party. The main data extracted are as follows: title, author, year, fund source, sample size, age, gender, duration of disease, interventions, outcome measures, adverse reactions, etc. If you find something unclear in the study, you can contact the author of the communication directly for more detailed information. The above information was finally cross-checked by 2 reviewers.

#### 2.4.3 Assessment of risk of bias in included studies

The quality assessment of RCTs adopts the risk of bias (ROB) assessment tool provided by the *Cochrane Handbook*. The following 7 items, such as random sequence generation, allocation concealment, blinding of participants and personnel, blinding of outcome assessment, incomplete outcome data, selective outcome reporting, and other bias, are evaluated by 3 grades of “low bias,” “high bias,” and “unclear bias.” The discrepancies will get a consistent conclusion by discussing between both reviewers or seeking the third-party consultation.

#### 2.4.4 Measures of treatment effect

Different evaluation methods are selected according to the different efficacy indicators. For the dichotomous data, we will choose the effect scale indicator relative risk (RR) with 95% confidence interval (CI) to represent. While the continuous data is expressed as mean difference (MD) or standardized mean difference (SMD) with 95% CI depending on whether the measurement scale is consistent or not.

#### 2.4.5 Dealing with missing data

The reviewers will contact the first author or correspondent author via email or telephone to obtain missing data if the relevant data is incomplete. If the missing data is still not obtained in the above way, we can synthesize the available data in the initial analysis. Furthermore, sensitivity analysis will be used to assess the potential impact of missing data on the overall results of the study.

#### 2.4.6 Assessment of heterogeneity

Heterogeneity will be assessed by Chi-squared test and *I*^2^ test. If *I*^2^ < 50%, *P* > .1, we consider that no statistical heterogeneity between each study and choose fixed effect model (FEM) to synthesize the data. If *I*^2^ ≥ 50%, *P* < .1, indicating that there is a statistical heterogeneity, the data is integrated by the random effect model (REM). In addition, due to differences in heterogeneity, we will conduct subgroup or sensitivity analysis to look for the potential causes.

#### 2.4.7 Data analysis

Review Manager software version 5.3 provided by the Cochrane Collaboration will be performed for data synthesis and analysis. The dichotomous data is represented by RR, continuous data is expressed by MD or SMD. If there is no heterogeneity (*I*^2^ < 50%, *P* > .1), the data is synthesized using a fixed effect model. Otherwise (*I*^2^ ≥ 50%, *P* < .1), a random effect model is used to analyze. Then subgroup analysis will be conducted based on the different causes of heterogeneity. If a meta-analysis cannot be performed, it will be replaced by a general descriptive analysis.

#### 2.4.8 Subgroup analysis

If the results of the study are heterogeneous, we will conduct a subgroup analysis for different reasons. Heterogeneity is manifested in the following several aspects, such as race, age, gender, different intervention forms, pharmaceutical dosage, treatment course.

#### 2.4.9 Sensitivity analysis

Sensitivity analysis is mainly used to evaluate the robustness of the primary outcome measures. The method is that removing the low-level quality study one by one and then merging the data to assess the impact of sample size, study quality, statistical method, and missing data on results of meta-analysis.

#### 2.4.10 Grading the quality of evidence

In this systematic review, the quality of evidence for the entire study is assessed using the “Grades of Recommendations Assessment, Development, and Evaluation (GRADE)” standard established by the World Health Organization and international organizations.^[32]^ To achieve transparency and simplification, the GRADE system divides the quality of evidence into 4 levels: high, medium, low, and very low.

## 3 Discussion

NAFLD has emerged a major challenge because of its prevalence, and lack of approved therapies. Documented evidence showing that NAFLD is a multisystem disease affecting multiple extrahepatic organ systems and interacting with the regulation of several metabolic/endocrine and pro-inflammatory pathways.^[33]^ Indeed, convincing evidence manifesting that NAFLD is strongly associated with type 2 diabetes.^[34]^

Vitamin D has gained increasing attention in different research fields. The vitamin D receptor (VDR) may play a role in the mechanisms for the link between vitamin D deficiency and other disorders, such as diabetes,^[35]^ nonalcoholic liver disease.^[36]^ Recent studies have displayed that vitamin D has a key role in the regulation of oxidative, the production of pro-inflammatory cytokines, hepatocyte apoptosis, and even liver fibrosis.^[37–39]^ It should be noted that vitamin D deficiency and NAFLD are also associated with IR and T2DM. For sure that NAFLD strongly related to T2DM, and both diseases are characterized by pro-atherogenic dyslipidemia, defined by hypertriglyceridemia, low high-density lipoprotein cholesterol concentrations, and the predominance of small dense LDL. In that low vitamin D levels are positively associated with NAFLD and T2DM. But there is absent convincing evidence-based medicine to confirm the efficacy of vitamin D supplementation for T2DM with NAFLD. Thus, we attempt to conduct this meta-analysis to analysis and summarize the efficacy of vitamin D supplementation for T2DM with NAFLD.

There are strengths in our study. Firstly, this meta-analysis provides a comprehensive assessment to whether vitamin D supplementation is beneficial for T2DM combined with NAFLD. Secondly, this study will provide clear evidence that vitamin D supplementation is good for diabetic patients with NAFLD. Moreover, RCTs will be included in our studies and appear to be high quality and low risk of bias. However, there may be some limitations in our meta-analysis. Firstly, both Chinese and English forms of research may increase the bias of the study. Secondly, the variety of race, age, gender, intervention forms, pharmaceutical dosage, and treatment course may result in higher clinical and statistical heterogeneity.

In conclusion, this study will help to determine the beneficial effects on diabetic patients with NAFLD. We hope this study will provide higher quality evidence for the benefits of vitamin D supplementation for T2DM combined with NAFLD.

## Author contributions

**Conceptualization:** Shengju Wang, Baochao Cai.

**Data curation:** Shengju Wang, Baochao Cai, Xuke Han.

**Formal analysis:** Xuke Han, Yang Gao.

**Funding acquisition:** Qiu Chen.

**Methodology:** Shengju Wang, Baochao Cai, Xuke Han.

**Project administration:** Xiaoran Zhang.

**Resources:** Shengju Wang, Baochao Cai, Ruili Wang.

**Software:** Shengju Wang, Baochao Cai, Yuan Zhang.

**Supervision:** Qiu Chen.

**Writing – original draft:** Shengju Wang, Baochao Cai.

**Writing – review & editing:** Qiu Chen.
